# Pediatric Dental Fluorosis and Its Correlation with Dental Caries and Oral-Health-Related Quality of Life: A Descriptive Cross-Sectional Study among Preschool Children Living in Belagavi

**DOI:** 10.3390/children10020286

**Published:** 2023-02-01

**Authors:** Vaibhav Kumar, Ridhima Gaunkar, Jasleen Thakker, Anil V. Ankola, Mamata Iranna Hebbal, Atrey J. Pai Khot, Virinder Goyal, Amel Ali, Elzahraa Eldwakhly

**Affiliations:** 1 Department of Public Health Dentistry, TPCT’s Terna Dental College, Nerul, Navi Mumbai 400706, Maharashtra, India; 2 Department of Public Health Dentistry, KLE Academy of Higher Education and Research, Vishwanath Katti Institute of Dental Sciences, Nehru Nagar 590010, Belagavi Karnataka, India; 3 Department of Public Health Dentistry, Government Dental College and Hospital, Bambolim 403202, Goa, India; 4 TPCT’s Terna Dental College, Nerul, Navi Mumbai 400706, Maharashtra, India; 5 Department of Preventive Dental Sciences, College of Dentistry, Princess Nourah Bint Abdulrahman University, P.O. Box 84428, Riyadh 11671, Saudi Arabia; 6 Guru Nanak dev Dental College and Research Institute, Sunam 148028, Punjab, India; 7 Department of Clinical Dental Sciences, College of Dentistry, Princess Nourah Bint Abdulrahman University, P.O. Box 84428, Riyadh 11671, Saudi Arabia

**Keywords:** dental fluorosis, deciduous dentition, oral-health-related quality of life, preschool children, Early Childhood Oral Health Impact Scale (ECOHIS)

## Abstract

Aim: This study aims to assess the prevalence of dental fluorosis and its association with dental caries, oral health behaviors, oral-health-related quality of life and parents’ perceptions among preschool children in the 3–5-year-old preschool children living in the Belagavi district of Karnataka, a non-endemic fluorosis region. Methods: A descriptive cross-sectional questionnaire-based study was conducted among 1200 individuals of the preschool population from 48 government-sponsored child-care development centers in Belagavi, Karnataka, over a three-month period. They were examined following the Dean’s fluorosis index (1942) and dmft (decayed, missed, and filled) scores of the participants were also recorded. Parents’ perception of oral health was assessed using the self-administered Early Childhood Oral Health Impact Scale (ECOHIS). SPSS software (version 20) was used for statistical analysis. Chi-square test computed categorical data. One-way ANOVA test was used for multiple group comparisons. *p* < 0.05 was considered statistically significant. Results: Of the 1200 children examined, 10 (0.83%) children exhibited dental fluorosis. Of the 10 children having fluorosis, six had fluorosis present on two or more of their primary teeth, and four had fluorosis on four or more teeth. The mean dmft score was 3.01 ± 1.38 to 3.60 ± 1.72 in 3–5-year-old children, respectively, with statistical significance difference (*p* < 0.001). The mean score of oral health-related quality of life was 10.74 ± 2.06, which was significantly correlated to the child’s age and parents’ education level (*p* < 0.05). Conclusion: The study shows a negligible amount of prevalence of dental fluorosis in the non-endemic fluorosis residential district. It also elucidates that children from lower and lower-middle socioeconomic status are more prone to suffer from dental fluorosis as compared to other groups. The mean score of ECOHIS increased proportionally with the caries experience, indicating a significant relationship between the dmft and ECOHIS score. Deciduous dentition fluorosis is often neglected, especially in areas that are not considered endemic for fluorosis and with only optimum fluoride levels in their groundwater, which supports the disease’s nature as multi-factorial and shows that a broader perspective is of prime importance to assess, diagnose, and prevent dental fluorosis among the preschoolers, thereby appraising their overall health and hygiene status.

## 1. Introduction

Fluoride has emerged as an anomalous arch-criminal, a double-edged sword revolving around oral hygiene and prevention. While its stint in the impediment of dental caries is in-grained, dental fluorosis, a well-known developmental enamel defect due to excessive fluoride ingestion during enamel formation, which is generally from chronic, long-term exposure to elevated levels of fluoride as a repercussion, is much unaccounted for [1]. Dental fluorosis reflects tooth development; therefore, excessive fluoride ingestion during early maturation and the secretory stage of enamel formation alters protein metabolism, producing a disorganized crystal structure and hypo-mineralization [2]. Primary dentition’s fluorosis is “rare” or “less severe” when compared to permanent dentition’s fluorosis. This is attributed to the placental tissue acting as a controlling factor on the concentration of fluoride in the fetal blood and the consumption of mother’s breast milk in which fluoride concentration is approximately 0.02 parts per million. Fluorosis of the primary dentition is considered to be of little consequence due to the temporary status of this dentition [3]. However, dental fluorosis when affecting the primary dentition had a prevalence of 29 percent in the optimal-fluoride area and 14 percent in the low-fluoride area, with a greater propensity to involve the succedaneum dentition by 1.86 times [4,5]. This concludes that primary dentition is equally if not more prone to dental fluorosis than permanent teeth. Proper diagnosis of dental fluorosis in the primary dentition avoids damage to the permanent successors both aesthetically and functionally. Deciduous teeth act as alarms for dental fluorosis, providing a basis for intervention [6]. The past 40 years have led to an array of sources of ingested fluoride, encompassing intentional sources such as foods, beverages, and dietary supplements, and unintentional sources of fluoride such as ingestion from fluoride dentifrices, mouth rinses, and other topical fluoride products. These factors coupled with malnutrition and childhood infections can substantially escalate the prevalence of dental fluorosis in preschool children. High-fluoride-level (1000 ppm or more) toothpastes administered to children under 3 to 5 years of age are linked with an increased risk of dental fluorosis. Approximately 0.1 mg of fluoride is usually consumed during use of toothpastes and mouth rinses, whereas 20 mg or more is ingested during professional fluoride gels applications. The major source of dietary fluoride is fluoridated water. A wide array of other sources of fluoride encompasses foods, beverages, and dietary supplements. Levy et al. reported that total fluoride intake, mostly from water, between the ages of 6 and 9 months was associated with fluorosis in the primary second molars [1,5,6].

Clinical measures used to quantify oral diseases include the decayed, missing, and filled index (dmft) for detection of caries or the Dean’s index for fluorosis [7]. The severity of clinical conditions such as caries and fluorosis influences children quality of life. The diversity in the influence of dental fluorosis on the quality of life of children is associated with different aesthetics perceptions as affected by the intensity of enamel mottling [7,8]. As well as causing discomfort in a significant population of children, dental caries interferes with body growth, negatively affecting body weight and height. As parents are responsible for their children health, assessing parents’ perceptions about their children quality of life is crucial. The Early Childhood Oral Health Impact Scale (ECOHIS) is used to assess the oral-health-related quality of life in children [9].

Fluorosis is an uprising public health problem for some residential zones such as the Indian subcontinent since it lies in the geographical fluoride belt that extends from Turkey to China and Japan through Iraq, Iran, and Afghanistan. According to the U.S. Center for Disease Control and Prevention (CDC), when the fluoride level of drinking water exceeds 1.5–2.0 ppm, the risk of developing fluorosis is enhanced, especially in children aged less than 8 years old. Sixty-two million people in India are affected by dental, skeletal, and non-skeletal fluorosis, out of which six million are children aged below 14 years old [10]. In Karnataka, several residential districts were noted with a range of fluoride concentration varying from 0.2 to 18.0 mg/L [3]. The permissible optimum fluoride level in groundwater was reported to be less than 1.5 mg/L by the Central Ground Water Board—Government of India, Ministry of Water Resources. Even with the presence of such optimum fluoride levels in residential zones, the pervasiveness of dental fluorosis was evident [11].

The empirical data on fluorosis and its association with dental caries and quality of life represent a vital research topic, as this affects a wide array of the pediatric population. Hence, the current study investigates dental fluorosis and its association with dental caries, oral health behaviors, oral-health-related quality of life, and parents’ perceptions among preschool children in a non-endemic fluorosis region.

## 2. Materials and Methods

A descriptive cross-sectional study was performed to assess dental fluorosis among 3–5-year-old children attending government-sponsored child-care development centers that are considered the first step of formal education, particularly for those from disadvantaged sections of society [12].

### 2.1. Sample Size Calculation and Sampling Technique

Using the following formula, the sample size was 1135, which was rounded off to 1200.


$$\begin{array}{l} \mathrm{Sample}\;\mathrm{Size}=4\mathrm{pq}/d^{2}\\ =4\times (77\times 23)/{\left(2.5\right)}^{2}\\ =1135 \end{array}$$

Prevalence of Dental Fluorosis: (p) = 77%;
Free of Dental Fluorosis: (q) = (1 − p) = 23%;
Absolute Admissible Error: (d) = 2.5%.

Written informed consent was obtained from the parents of all the participating preschool children.

Inclusion criteria:

1. Children 3–5 years of age attending government-sponsored child-care development Centers at Belagavi;
2. Children whose parents were willing to give written informed consent.

Exclusion criteria:

1. Physically or medically compromised children;
2. Children with debilitating diseases or suffering from uncontrolled systemic conditions.

This study was conducted during the period from June to August, and data were collected over the duration of three months in the district of Belagavi, Karnataka, which consists of 1208 villages according to the official government documents. A multistage sampling method was employed in which 60 random villages were selected through computer-generated randomization. From those 60 villages, 20 children from each government-sponsored child-care development center were selected through simple randomization of the list of children in the selected center.

The ethical clearance was obtained from KLE, Vishwanath Katti Institute of Dental Sciences Institutional Research and Ethics Committee, with reference number 903. An exempt status for the study was granted by the ethical committee, as it was decided that the study poses no risks to participants.

### 2.2. Clinical Examinations

Clinical examination was conducted in a well-ventilated room with adequate natural light. Dental mouth mirrors were used to visualize the oral cavity and dental status. Cotton rolls were used to isolate the teeth during examination. After extensive training and calibration of the examiners for examining fluorosis lesions, in order to present with minimal chances of missed dental fluorosis diagnosis, clinical examinations were performed. The principal investigator (V.K.) and the coinvestigator (R.G.) measured the dmft index (decayed, missing, and filled teeth) using dental examination tools (disposable dental mirror, dental explorer, sterile gauze, and mask) according to the World Health Organization criteria for caries diagnosis. A clerk recorded the findings and assisted the investigators in any possible way. Hence, the kappa coefficient value for intra-examiner reliability was determined and found to be 0.87. The standardized scoring was carried out by the Dean’s fluorosis index (1942). The buccal, lingual, and occlusal surfaces were examined.

### 2.3. Questionnaires and Data Collection

#### 2.3.1. Development of the Questionnaire

The questionnaire was adopted from Pahel BT et al. [13] with minor modifications to incorporate oral hygiene practices and make the questionnaire more apprehensible to parents. Four experts with extensive research in maternal and child health reviewed the contents of the questionnaire based on validity assessment. The questionnaire was translated by two experts. The first expert carried out translation from English to Kannada, after which the second expert back translated it to English. The Kannada-translated English version was compared with the original English version to confirm whether the words were similar without a change in the meaning. The questionnaire was then distributed among five participants to assess their understanding of the words. The reliability of the questionnaire was checked by randomly selecting six participants to answer the questionnaire for two times at two separate time intervals. The mean Lawshe’s content validity ratio was discerned to stand at 0.89.

#### 2.3.2. Details of the Questionnaire

The self-administered questionnaire (ECOHIS) consisted of 12 questions that examined the parent’s perception regarding the impact of their children’s oral health on their quality of life. The questions targeted the knowledge of parents regarding their children’s oral and dental pain, troubles during eating and drinking, difficulty in pronunciation, inability to sleep, irritation or frustration, avoidance of talking or smiling, cleaning of teeth and gums, rinsing after every meal, brushing twice daily, the quantity of toothpaste and supervised tooth brushing, toothbrush changing, and dentist check-up visits. The responses were graded as “never”, “hardly ever”, “occasionally”, “often”, and “very often”, with corresponding scores of 0 to 5, respectively. A score for the missing items was considered as an average of the remaining items for each section. Collectively, the entire score of this index ranged from zero to sixty. The higher the recorded total score, the greater the number of reported oral health problems and the poorer the oral-health-related quality of life.

#### 2.3.3. Data Collection Procedure

The significance of conducting the study was explained to the parents, and their informed consent was taken before participation in the study. Parents were requested to choose the best sensible presented option to answer each question, and adequate time was allowed to complete answering the questionnaire. Confusions were clarified to parents without hinting at the correct answer. To gather data regarding the socio-economic status of the participating families, the Kuppuswamy’s socio-economic scale was used, whereby information regarding the education and occupation of the head of the family along with monthly income were collected. Data obtained were scored in the range of 3 to 29, classifying the household as having a high, middle, and low socio-economic status. An interactive oral health education camp was plotted based on the collected data. Affected children were referred to a dental hospital for comprehensive treatment.

### 2.4. Statistical Analysis

Data were statistically analyzed using IBM Corp., released in 2011. IBM SPSS Statistics for Windows, Version 20.0. Armonk, NY: IBM Corp. Descriptive statistics were calculated. Frequency, percentages for categorical data, and means and standard deviations were recorded. The chi-square test, Independent *t*-test, and Spearman’s correlation were exploited for statistical analysis. The significance level was set at *p* < 0.05.

## 3. Results

A total of 10 (0.83%) of the 1200 children examined exhibited dental fluorosis. Of the ten children having fluorosis, six children presented fluorosis on two or more primary teeth, and four children showed fluorosis on four or more primary teeth. The sociodemographic characteristics of participants with dental fluorosis are given in Table 1.

The mean dmft score was substantially lesser in participants exhibiting dental fluorosis, Table 2.

The occurrence of fluorosis was noted bilaterally in one or both arches, so an even number of affected teeth was presented in each child. The mean number of affected teeth was 4.21 ± 1.35. The fluorosis in nearly all teeth was mild (Dean’s index criteria = 3). Only two individuals reported higher Dean’s scores (Dean’s index criteria = 4). The primary second molar teeth seemed to be the most affected (90%), followed by the first molars (40%). Canines and incisors were the least affected. The buccal surface was the most affected surface of second molar teeth with fluorosis in nearly 80% of cases, whereas the occlusal and lingual surfaces were affected in only about 25–35% of cases. Moreover, 76% to 92% of affected second molar teeth exhibited fluorosis on the gingival third of the buccal surfaces, whereas 26 to 48% of affected second molar teeth displayed fluorosis on the middle and occlusal thirds of the buccal surfaces.

The migrant subjects (*p*-value < 0.001), those belonging to lower and lower-middle socioeconomic status (*p*-value = 0.004), children who were in a government-sponsored child-care development center for one year (*p*-value < 0.001), and those with a starting age of tooth brushing with fluoridated toothpaste less than two years (*p*-value = 0.003) exhibited greater fluorosis. Regression analysis exhibited that migrants were 2.43 times more likely to have dental fluorosis. Brushing more than two times a day was 1.23 times more likely to cause dental fluorosis (Table 3).

The mean score of the oral-health-related quality of life with gender distribution was 10.74 ± 2.06, which indicated poorer oral health in children according to parents’ perceptions. The ECOHIS scale in domain of dental pain (17%), trouble sleeping (13.92%), irritability, and frustration (10.75%) with gender as independent factor was found to be statistically significant (*p* ≤ 0.001). Parents’ perceptions regarding their child’s teeth and gums cleaning, rinsing after every meal, the quantity of toothpaste used, frequency of brushing and changing the tooth brush as definitively affecting their child’s oral-health-related quality of life was statistically significant (*p* ≤ 0.001) (Table 4).

The correlation between dmft scores and fluorosis scores with ECOHIS scores was statistically significant (r = 0.375, *p* ≤ 0.001 for the dmfd) (r = −0.142, *p* = 0.028 for the Dean’s fluorosis index), indicating that the caries experience has a robust influence on the quality of life of a child (Table 5).

## 4. Discussion

The impending oral health status of an adult is broadly established in the preschool period, and more than 90% of such dental diseases, with timely diagnosis and prevention ventured around this age, could be prevented, as stated by Kumar et al. [12]. For this very reason, the Indian government has initiated a module, the Integrated Child Development Scheme (ICDS), targeting children under six years of age, pregnant women, and nursing mothers. The program intends to supplement basic health services via a government-sponsored child-care development center for a population of 1000 people with the responsibility of organizing informal preschool education in the government-sponsored child-care development center for children belonging to the 3–5-year-old group [14]. Due to the scarcity of epidemiological data on fluorosis prevalence in deciduous dentition, the current study incorporated an inclusive oral health status assessment of preschoolers, focusing on dental fluorosis in the Belagavi district of Karnataka, which is proven to exhibit optimum fluoride levels, as a representative for residential regions with non-endemic fluorosis.

In the present study, of the 1200 children examined, 0.83% of children exhibited dental fluorosis. These results were in contrast with the study from C.B. Shivayogimath et al. [15], which reported a prevalence of 5.56% dental fluorosis in a similar non-endemic fluorosis residential region with a greater fluoride level in their groundwater (0.84 ppm) as compared to 0.48 ppm for this study’s non-endemic fluorosis residential region (Belagavi District) [16]. Another study by N. Divyalalitha et al. [17], conducted amongst children attending other government-sponsored child-care development centers, reported that a 0.4% dental fluorosis rate could be credited to the fact that the area where this study was conducted was non-fluorosis-prone. This could be compared to a much more significant value of 3.40% dental fluorosis incidence in other residential zones with reported higher fluoride concentrations (4.22 mg/L) in groundwater samples [9].

Under the scenario found in the present study, those belonging to lower and lower-middle socioeconomic status (16.7%) and mothers with a comparatively lower level of education (1.24%) were majorly prone to fluorosis, which is in line with the conclusion drawn by Pontigo-Loyola et al. [18]. A potential explanation could be that lower and lower-middle socioeconomic parents showed deficient health knowledge regarding the probable harm of fluorides if ingested in excessive amounts. Likewise, lower and lower-middle socioeconomic parents were more likely to use water directly from springs or wells with increased fluoride levels. In addition, the use of fluoridated dentifrices with about 1 mg of fluoride per gm of dentifrice could have a potential role in increasing dental fluorosis. Children from 2–5 years of age ingest an average of 48 percent of this 1 mg of fluoride per gm of dentifrice, so it is apparent that unsupervised early childhood tooth brushing substantially increases fluoride exposure [19]. In our study, this effect could be confirmed since preschoolers brushing more than two times a day and applying a full brush of paste showed an increased risk of acquiring dental fluorosis.

Of the 0.83% of children with fluorosis in our study, 60% of them displayed fluorosis on two or more of their deciduous teeth, and 40% displayed fluorosis on four or more deciduous teeth. The average number of teeth affected by fluorosis was 4.21 ± 1.35. These results were in congruence with pioneer studies conducted by A Thylstrup et al. [20], Levy et al. [2], Sami et al. [21], and John J Warren et al. [22] to evaluate the distribution of dental fluorosis in the deciduous dentition. The statistically significant similarity among the studies could be narrowed down to potential risk factors attributing to a greater prevalence of fluorosis noted in the second molars as compared to the anterior teeth. The scarce incidence of dental fluorosis among the deciduous incisors is attributed to the placental tissues acting as a regulating factor for the concentration of fluoride in the fetal blood [2]. The greater prevalence of dental fluorosis on the gingival third of the primary second molars may be attributed to postnatal fluoride exposure. As infants mature, their diets become more varied, and the fact that fluorosis occurs in children residing in areas with low fluoride levels could be as a result of ingestion of dietary fluoride sources other than water [23]. At the age group of 3–5 years, the mother stops breastfeeding the child and is inclined towards providing milk through infant formulas and powdered milk, which contributes to a higher incidence of dental fluorosis in the post-natal period [24]. Osuji et al. confirmed that prolonged use of powdered milk for 13 months or more increased the fluorosis rate [6].

Dental fluorosis and dental caries are two variables that often go hand in hand, and while a study by Van Nieuwenhuysen et al. [25] speculated on low caries prevalence in subjects with greater fluorosis, our current study also shows a reduction in caries, with the mean dmft score being substantially lower in participants exhibiting dental fluorosis. A child’s caries experience is associated with presence of oral symptoms and/or functional confines [16]. Children who exhibited mild fluorosis showed better perception of oral health with all other factors being controlled for multivariate models.

Responding to the ECOHIS questionnaire was completely dependent on parents’ apprehension of their children’s health and disease status. Accordingly, besides social aspects, parents’ education level had a major effect on the study results. Children’s lack of cognitive growth was a significant challenge during evaluation of their dental and oral disease. Consequently, parents were in charge of assessment of their children’s quality of life. According to the current study’s findings, the oral-health-related quality of life mean score (10.74 ± 2.06) was higher compared to studies by Sajadi et al. [26] (4.07 ± 0.79) and Scarpelli et al. [27] (2.6 ± 3.3). The results from the current study indicated that oral disease affects child socialization, self-confidence, and even learning abilities [26]. Furthermore, correlation between ECOHIS scores and dental disease experience confirms that parents are capable of providing effective feedback regarding their preschool children’s oral-health-related quality of life. Furthermore, a study by Sakaryali et al. [28] indicated that both simple and severe dental caries result in aesthetic and functional problems in children as well as imposing a negative impact on parents’ daily life.

Preschool children attending the government-sponsored child-care development centers form an ingrained entity entitled to focused intervention pertaining to oral health hygiene and other important constructs of overall general well-being. This hypothesis-generating descriptive study opens channels for such oral-health-related translational activities to be designed, implemented, and periodically evaluated as part of the standard procedures and protocols of health care maintenance. Parents should appreciate the importance of maintaining good health of primary teeth and how the oral health status of the child affects his/her quality of life and thereby instill a positive attitude towards preventive and therapeutic dental aid. Following a need-based assessment, governments should allocate funds to support oral health and hygiene enhancement in government-sponsored child-care development centers in regions prone to fluorosis.

### Limitations of the Study

The current study was carried out in only one non-endemic fluorosis region, and thus, the selected study population does not precisely embody the whole population. Further studies in similar non-endemic regions should be conducted to compare and contrast the results obtained to better understand the influence on fluorosis in the primary dentition. Even during the sampling of the study, areas that might have higher fluorosis prevalence data could have been missed due to the randomized assortment of the residential districts to be included in the study. Henceforth, this study’s results should be generalized with caution.

## 5. Conclusions

The study shows a negligible amount of prevalence of dental fluorosis in less-endemic-prone areas. It also shows that children from lower and lower-middle socioeconomic status are more prone to suffer from dental fluorosis as compared to other groups. The mean score of ECOHIS increased proportionally with the dmft index score, demonstrating a significant association between the dmft and ECOHIS score. These findings can serve as an appropriate resource for the development of preventive programs and the promotion of young children’s oral health.

## Figures and Tables

**Table 1:** Sociodemographic characteristics of children with enamel fluorosis.

	Number of Participants	Enamel Fluorosis	*p*-Value
**Age**			
3 years	493 (41.08)	3 (0.6)	0.106
4 years	518 (43.17)	3 (0.58)	
5 years	189 (15.75)	4 (2.1)	
**Gender**			
Male	589 (49.08)	6 (1.01)	0.488
Female	611 (50.92)	4 (0.7)	
**Socio-economic status**			
Upper	36 (3)	0	0.004 *
Upper middle	175 (14.58)	0	
Lower middle	216 (18)	5 (13.8)	
Lower	773 (64.42)	5 (2.9)	
**Migration status**			
Non-migrant	923 (76.92)	2 (0.22)	≤0.001 *
Migrant	277 (23.08)	8 (2.9)	
**Duration in government-development center**			
1 year	505 (42.08)	10 (100)	≤0.001 *
2 years	506 (42.17)	0	
3 years	189 (15.75)	0	
**Level of education of mother**			
Primary school	644 (53.67)	8 (1.24)	0.093
High school	556 (46.33)	2 (0.36)	
**Age of stating tooth brushing**			
<2 years	527	9 (1.71)	0.003 *
>2 years	673	1 (0.15)	

Chi-square test is used, and values are expressed as frequency with percentages (in parentheses); * *p* ≤ 0.05 denotes statistical significance.

**Table 2:** The dmft status of study population.

Variable Group	Mean ± SD	*p*-Value
dmft, no fluorosis	3.24 ± 1.75	0.001 *
dmft, fluorosis	0.03 ± 0.02	

Independent *t*-test is used. Values are expressed as mean ± standard deviation (SD). * *p* ≤ 0.05 denotes statistical significance.

**Table 3:** Regression analysis to predict the outcome dental fluorosis in primary dentition.

		Number of Subjects	Enamel Fluorosis	Non-Enamel Fluorosis	Relative Risk	Odds Ratio (OR)	*p*-Value
**Migration status**	Non-migrant	923	2	921	0.08	1 ^a^	0.001 *
	Migrant	277	8	269	13.15	2.43	
**Brushing frequency**	≥2 times/day	683	10	673	0.015	1.23	0.002 *
	<2/day	517	0	517		1 ^a^	

Regression analysis is used. 1 ^a^: reference; level of significance: * *p* ≤ 0.05 denotes statistically significant correlation, absence of risk and ratio is represented by grey sections.

**Table 4:** ECOHIS self-administered questionnaire responses of parents’ perception about oral health of children (N = 1200).

Parents Perception	Never N (%)	Hardly Ever N (%)	Occasionally N (%)	Often N (%)	Very Often N (%)	*p*-Value
Oral/dental pain	486 (40.5)	227 (18.92)	126 (10.5)	157 (13.08)	204 (17)	0.0044 *
Male	283 (48.05)	59 (10.02)	77 (13.07)	65 (11.03)	105 (17.83)	
Female	203 (33.22)	168 (27.49)	49 (8.02)	92 (15.06)	99 (16.20)	
Difficulty eating and drinking	829 (69.08)	215 (17.92)	71 (5.92)	65 (5.42)	20(1.67)	0.083
Male	481 (81.66)	36 (6.11)	16 (2.72)	40 (6.79)	16 (2.72)	
Female	348 (56.95)	179 (29.30)	55 (9.00)	25 (4.09)	4 (0.65)	
Difficulty pronouncing words	963 (80.25)	105 (8.75)	28 (2.33)	58 (4.83)	46 (3.83)	0.533
Male	459 (77.93)	59 (10.02)	18 (3.06)	27 (4.58)	26 (4.41)	
Female	504 (82.49)	46 (7.53)	10 (1.64)	31 (5.07)	20 (3.27)	
Trouble sleeping	213(17.75)	262(21.83)	377(31.42)	181(15.08)	167(13.92)	≤0.001 *
Male	111 (18.84)	96 (16.30)	254 (43.12)	75 (12.73)	53 (8.99)	
Female	102 (16.69)	166 (27.17)	123 (20.13)	106 (17.35)	114 (18.66)	
Irritable or frustrated	398 (33.17)	316 (26.33)	150 (12.5)	207 (17.25)	129 (10.75)	≤0.001 *
Male	186 (31.58)	169 (28.69)	62 (10.53)	109 (18.50)	63 (10.70)	
Female	212 (34.70)	147 (24.96)	88 (14.40)	98 (16.04)	66 (10.80)	
Avoids talking or smiling or laughing	533 (44.42)	245 (20.42)	224 (18.67)	92 (7.67)	106 (8.83)	0.067
Male	284 (48.22)	101 (17.15)	124 (21.05)	36 (6.11)	44 (7.47)	
Female	249 (40.75)	144 (23.57)	100 (16.37)	56 (9.16)	62 (10.15)	
Child’s teeth and gums cleaning	27 (2.25)	189 (15.75)	363 (30.25)	438 (36.5)	183 (15.25)	≤0.001 *
Male	15 (2.55)	72 (1.22)	184 (31.24)	232 (39.39)	86 (14.60)	
Female	12 (1.96)	117 (19.15)	179 (29.30)	206 (33.71)	97 (15.87)	
Rinsing child’s mouth after every meal	192 (16)	284 (23.67)	305 (25.42)	260 (21.67)	159 (13.25)	0.368
Male	78 (13.24)	164 (27.84)	113 (19.18)	146 (24.79)	88 (14.94)	
Female	114 (18.66)	120 (19.64)	192 (31.42)	114 (18.66)	71 (11.62)	
Brushing twice daily	56 (4.67)	276 (23)	439 (36.58)	211 (17.58)	218 (18.17)	≤0.001 *
Male	18 (3.06)	145 (24.62)	229 (38.88)	96 (15.71)	101 (17.15)	
Female	38 (6.22)	131 (21.44)	210 (34.37)	115 (18.82)	117 (19.15)	
Quantity of toothpaste supervised	351 (29.25)	454 (37.83)	295 (24.58)	86 (7.17)	14 (1.17)	≤0.001 *
Male	168 (28.52)	241 (40.92)	137 (23.26)	38 (6.45)	5 (0.85)	
Female	183 (29.95)	213 (34.86)	158 (25.86)	48 (7.85)	9 (1.47)	
Changing the toothbrush	265 (22.08)	337 (28.08)	226 (18.83)	197 (16.42)	175 (14.58)	0.293
Male	136 (23.09)	138 (23.43)	134 (22.75)	89 (15.11)	92 (15.62)	
Female	129 (21.11)	199 (32.57)	92 (15.06)	108 (17.67)	83 (13.58)	
Dentist visit for check up	368 (30.67)	218 (18.17)	207 (17.25)	238 (19.83)	169 (14.08)	0.062
Male	190 (32.26)	116 (19.69)	85 (14.43)	115 (19.52)	83 (14.09)	
Female	178 (29.13)	102 (16.69)	122 (19.97)	123 (20.13)	86 (14.07)	
Overall Mean ± SD ^#^	10.74 ± 2.06					

Chi-square test is used, and values are expressed as frequency with percentages (in parentheses); * *p* ≤ 0.05 denotes statistical significance; # overall mean is obtained by calculating total ECOHIS score per child.

**Table 5:** Correlation between the variables.

		Dmft Index Scores	Dean’s Fluorosis Index Scores
**ECOHIS score**	Correlation coefficient (rho)	0.375	−0.142
	Sig. (2-tailed) *p*-value	≤0.001 *	0.028 *

Spearman’s correlation is used; * *p* ≤ 0.05 denotes statistical significance.

## Data Availability

The data presented in this study are available on request from the corresponding author.
